# Systemic Immune-Inflammation Index and Changes of Neutrophil-Lymphocyte Ratio as Prognostic Biomarkers for Patients With Pancreatic Cancer Treated With Immune Checkpoint Blockade

**DOI:** 10.3389/fonc.2021.585271

**Published:** 2021-02-24

**Authors:** Jin Shang, Xiao Han, Haoran Zha, Haitao Tao, Xiaoyan Li, Fang Yuan, Guangying Chen, Lijie Wang, Junxun Ma, Yi Hu

**Affiliations:** ^1^ Department of Oncology, Chinese People's Liberation Army General Hospital, People's Liberation Army School of Medicine, Beijing, China; ^2^ Department of Health Service, Guard Bureau of the Joint Staff Department, Central Military Commission of People's Liberation Army, Beijing, China; ^3^ Department of Oncology, People's Liberation Army Rocket Force Characteristic Medical Center, Beijing, China

**Keywords:** pancreatic cancer, immune checkpoint blockade, systemic immune-inflammation index, neutrophil-lymphocyte ratio, immunotherapy, prognosis

## Abstract

The efficacy of current treatment regimens for pancreatic cancer (PC) remains unsatisfactory. In recent years, immune checkpoint blockade (ICB) therapy has shown promising anti-tumor outcomes in many malignancies, including PC. Inexpensive and readily available biomarkers which predict therapeutic responses and prognosis are in critical need. Systemic immune-inflammation index (SII) and neutrophil-lymphocyte ratio (NLR) are emerging predictors for prognosis of various tumors. We aim to investigate the prognostic significance of baseline SII, NLR, and their changes in PC patients treated with ICB. Our retrospective analysis included PC patients treated with ICB therapy in the Chinese PLA General Hospital. All demographic, biological, and clinical data were extracted from medical records. Relative changes of SII after two doses of ICB were defined as ΔSII% and calculated as (SII_after 2 doses_-SII_baseline_)/SII_baseline_, and so was the case for ΔNLR%. Overall survival (OS) and progression-free survival (PFS) were compared using Kaplan-Meier curves. The prognostic significance of baseline SII, NLR, and their changes was assessed in univariate and multivariate analyses using the Cox proportional hazard regression model. In total, 122 patients with PC treated with ICB were included in the present analysis. Elevated baseline SII (HR=3.28; 95% CI:1.98–5.27; *P*=0.03) and ΔNLR% (HR=2.21; 95% CI:1.03–4.74; *P*=0.04) were significantly correlated with an increased risk of death. For PC patients receiving ICB combined with chemotherapies or radiotherapies as the first-line treatment, increased baseline SII was a negative predictor for both OS (HR=8.06; 95% CI:1.71–37.86; *P*=0.01) and PFS (HR=2.84; 95%CI:1.37–10.38; *P*=0.04). Our study reveals the prognostic value of baseline SII and NLR changes in PC patients receiving ICB therapy. The clinical utility of these prognostic biomarkers needs to be further studied in prospective studies.

## Introduction

Pancreatic cancer (PC) is a highly aggressive malignancy with one of the lowest survival rates among all tumor entities, with an overall 5-year survival rate of less than 5% (1). In recent years, there have been few breakthroughs in the early diagnosis and effective therapy for pancreatic cancer patients (2). The scarcity of early PC symptoms makes it very difficult to obtain an early diagnosis. Only 15% to 20% of all newly diagnosed patients have the opportunity for surgical resection (3), and most PC patients have to rely on chemotherapies and palliative care. Chemotherapies such as FOLFIRINOX and gemcitabine/nab-paclitaxel increase the median survival time by only 2**–**4 months while showing significant adverse effects (4, 5). In addition, the median progression-free survival (PFS) of PC patients with intensive poly chemotherapies is 6 months or below (6, 7). Thus, new effective therapeutic regimens are urgently needed.

In recent years, immune checkpoint blockade(ICB) has gradually become a promising alternative treatment for various cancers (8), including pancreatic cancer (9, 10). Until now, the ICB mostly targets cytotoxic T lymphocyte antigen 4 (CTLA-4) and programmed cell death receptor 1 (PD-1) (11, 12). As important components of immune homeostasis, immune checkpoints prevent overreaction of immune response and play vital roles in peripheral tolerance (13). However, tumor cells often “hijack” immune checkpoint signaling pathways to escape from immune surveillance (14). Thus, ICB therapy uses specific monoclonal antibodies to revive T cells from exhausted status and restore their anti-tumor response (15). However, there is still a notable portion of PC patients who don’t respond to ICB therapy. It’s important to identify those who are most likely to have better clinical outcomes after taking ICB treatment. Therefore, convenient prognostic biomarkers have great clinical significance.

Currently, cancer-associated inflammation, such as increased and defective myelopoiesis, as well as local and systemic inflammation, is found to be closely related to tumorigenesis, disease progression and clinical prognosis (16, 17). Systemic inflammation can be measured to some extent using readily available peripheral blood parameters, such as systemic immune-inflammation index (SII) and neutrophil-lymphocyte ratio (NLR). The SII is a combination of platelets, neutrophils and lymphocytes, and the prognostic significance of SII was firstly reported in hepatocellular carcinoma (18). Until now, the prognostic value of SII has been investigated in several solid malignancies, such as esophageal squamous cell carcinoma, gastric cancer, non-small-cell lung cancer, and colorectal cancer (19–22). Studies have shown that an increased NLR or CRP predicts poor clinical outcomes of PC patients treated with the chemotherapies and surgeries (23, 24). However, the association between peripheral blood biomarkers and survival in PC patients receiving ICB therapy has to our best knowledge not been confirmed.

Accordingly, the aim of this study is to investigate the prognostic significance of baseline SII, NLR and their changes in PC patients treated with ICB therapy.

## Materials and Methods

### Patients and Study Design

We performed a retrospective analysis of all patients pathologically or clinically diagnosed with advanced pancreatic cancer treated with ICB in the Department of Oncology, Chinese PLA General Hospital between January 2015 and December 2019. Written informed consent was obtained from all included patients. Patients who had autoimmune and chronic infectious diseases, or had an acute infection just before the first dose of ICB therapy, or took any medication likely to interfere with hematological parameters, were excluded from the study. Moreover, PC patients who received ICB as the postoperative adjuvant therapy were also excluded. The ICB therapy consisted of nivolumab, pembrolizumab, atezolimab, ipilimumab, and sintilimab.

The study protocol was reviewed and approved by the Ethics Committee of Chinese PLA General Hospital and was in accordance with the principles promulgated in the Declaration of Helsinki.

### Data Collection

Patients’ baseline demographic, biological, and clinical data, including age at the start of ICB therapy, gender, tumor stage, tumor location, ordinal line number of ICB therapy, body-mass index (BMI), Karnofsky Performance Status (KPS) scores, radical resection surgery, CA19-9, and serum lactate dehydrogenase (LDH), were extracted from medical records. The immune signature of interest included the white blood cell count (WBC), absolute neutrophil count (ANC), absolute lymphocyte count (ALC), absolute monocyte count (AMC) and hemoglobin (HGB). The baseline immune signature was obtained before the first dose of ICB treatment and after two doses of ICB therapy, the immune signature was once again evaluated. All the results were also extracted from medical records. The tumors were assessed at baseline and every 2 doses of ICB therapy. Tumor response after ICB therapy was classified according to a modified Response Evaluation Criteria in Solid Tumors 1.1 for immune-based therapeutics (iRECIST). The regular follow-up was performed every 3 months and was terminated on 31^st^ December, 2019. The PFS was defined as the time interval from the date of first dose of ICB therapy to progression or death due to any cause. The overall survival (OS) was defined as the time interval from the date of first dose of ICB therapy to death due to any cause.

### Statistical Analysis

The baseline characteristics of all patients were described and summarized using frequencies and percentages for categorical variables and medians and ranges for continuous variables. Differences were considered statistically significant at *P* < 0.05. The ratios were calculated using the formulas: NLR = ANC/ALC and SII=PLT×ANC/ALC. Relative changes in the SII were defined as ΔSII % and calculated as (SII _after 2 doses_- SII _baseline_)/SII _baseline_, and so was the case for ΔNLR%. The optimal cutoff values for prognostic factors were determined by the receiver operating characteristic (ROC) curve. The Kaplan-Meier method was utilized to compare the distributions of PFS and OS in different groups, and the differences were estimated using the log-rank test. The univariate and multivariate analyses were performed using the Cox proportional hazard regression model. Covariates that showed significant associations with survival (OS and PFS) in the univariable analysis were subjected to multivariable analysis which was based on a forward: LR procedure with enter and remove limits of 0.05 and 0.10, respectively. Results were presented as hazard ratios (HRs) with 95% confidence intervals (CIs). The proportional hazard assumption was evaluated using Shoenfeld residuals and no violation was found. All statistical analyses were conducted using SPSS software, version 26.0 (IBM, USA).

## Results

### Baseline Characteristics

In total, 122 PC patients were included in this study. Patient characteristics at baseline are summarized in **Table 1**. The median age of the patients at the start of the first ICB therapy was 56 years (range 33–85), and 71.3% were male. Only 11 (9.0%) patients had locally advanced PC at diagnosis, while 111 (91.0%) had metastatic PC. The tumor location of 61 (50.0%) patients was the head of the pancreas, while that of 54 (44.3%) was the body-tail of the pancreas and 2 patients had cancer at both locations. 45 patients (36.9%) received ICB therapy combined with chemotherapies as first-line treatment, 53 (43.4%) patients as second-line and 24 (19.7%) as third-line treatment or beyond. By the end of December 2019, 28 patients (23.0%) were confirmed to be alive, 49 (40.2%) had confirmed disease progression, and 21 (17.2%) were lost to follow-up. The median OS was 4.8 months (0.5–77.7) and median PFS was 3.3 months (0.3–44.7). A total of seven (5.7%) patients had neutrophilia (ANC>7.5×10^9^/L) before the start of ICB immunotherapy. Among all 122 patients, 68 patients were radiologically evaluable with the objective response rate of 14.7%, including one complete response and nine partial responses.

**Table 1:** Baseline characteristics.

Characteristic	Total (N = 122)
**Age, median (range)**	56 (33–85)
**Gender, N (%)**	
Male	87 (71.3)
Female	35 (28.7)
**Tumor stage at diagnosis, *N* (%)**	
locally advanced	11 (9.0)
metastatic	111 (91.0)
**Tumor location, *N* (%)**	
head	61 (50.0)
body-tail	54 (44.3)
both	2 (1.4)
unavailable	5 (4.3)
**Line of ICB treatment, *N* (%)**	
1	45 (36.9)
2	53 (43.4)
≥3	24 (19.7)
**CA19-9, u/ml, *N*(%)**	
<1,000	66 (54.1)
≥1,000	51 (41.8)
Unavailable	5 (4.1)
**Previous surgery, *N* (%)**	
yes	40 (37.5)
no	82 (62.5)
**KPS, *N* (%)**	
≥90	92 (75.4)
80	20 (16.4)
70	6 (4.9)
≤60	4 (3.3)
**BMI, *N* (%)**	
<18.5	30 (24.6)
18.5≤BMI<24	72 (59.0)
≥24	15 (12.3)
Unavailable	5 (4.1)
**LDH, *N* (%)**	
normal	72 (59.0)
abnormal	49 (40.2)
unavailable	1 (0.8)

ICB, immune checkpoint blockade; KPS, Karnofsky Performance Status; LDH, lactate dehydrogenase; BMI, body-mass index.

### Association Between Blood Biomarkers and Prognosis

#### OS

In univariate Cox regression model analysis, several factors, such as CA19-9, line of ICB treatment, ANC, SII, NLR, ΔSII%, and ΔNLR% were all significantly associated with OS (details in **Table 2**); while in multivariate analysis, only elevated CA19-9 (HR=2.52; 95% CI:1.21–5.23; *P*=0.01), AMC (HR=2.38; 95% CI:1.38–4.28; *P*=0.003), SII (HR=3.28; 95% CI:1.98–5.27; *P*=0.03) and ΔNLR% (HR=2.21; 95% CI:1.03–4.74; *P*=0.04) were significantly correlated with an increased risk of death (**Table 2**).

**Table 2:** Univariate and multivariate Cox regression analyses of prognostic factors associated with prognosis.

Factors	Overall survival				Progression-free survival			
	Univariate analysis		Multivariate analysis		Univariate analysis		Multivariate analysis	
	HR (95% CI)	*P*	HR (95% CI)	*P*	HR (95% CI)	*P*	HR (95% CI)	*P*
Gender								
male vs female	0.90 (0.55–1.49)	0.69			0.83 (0.53–1.31)	0.43		
Age								
≥ 65 vs < 65	1.21 (0.69–2.12)	0.50			0.98 (0.60–1.61)	0.93		
KPS scores		0.08				<0.001		
70 or 80 vs ≥ 90	1.30 (0.76–2.23)				1.45 (0.88–2.37)			
≤ 60 vs ≥ 90	3.35 (1.03–10.87)				6.12 (2.17–17.24)			
BMI		0.09				0.08		
BMI < 18.5 vs 18.5 ≤ BMI < 24	1.79 (1.05–3.04)				1.74 (1.07–2.83)			
BMI ≥ 24 vs 18.5 ≤ BMI < 24	1.07 (0.55–2.08)				1.23 (0.67–2.27)			
LDH								
abnormal vs normal	0.88 (0.55–1.42)	0.60			1.29 (0.85–1.95)	0.24		
Tumor stage								
metastatic vs locally advanced	3.15 (0.99–10.01)	0.05			3.79 (1.38–10.36)	0.005		
CA19-9								
≥1000 vs <1000 u/ml	2.60 (1.59–4.23)	<0.001	2.52 (1.21–5.23)	0.01	1.47 (0.96–2.25)	0.08		
Previous surgery								
no vs yes	1.36 (0.82–2.23)	0.23			1.44 (0.92–2.27)	0.11		
Line of ICB treatment		<0.001				<0.001		
2 vs 1	1.27 (0.73–2.22)				1.60 (0.98–2.62)			
≥3 vs 1	3.89 (2.07–7.30)				3.35 (1.89–5.93)			
WBC								
≥4.9 vs <4.9*10^9^/L	2.23 (1.32–3.77)	0.003			1.62 (1.04–2.53)	0.03		
ANC								
≥3.3 vs <3.3*10^9^/L	2.53 (1.53–4.18)	<0.001			2.01 (1.30–3.11)	0.001	3.07 (1.69–5.60)	<0.001
ALC								
≥1.1 vs <1.1*10^9^/L	0.48 (0.30–0.78)	0.003			0.61 (0.40–0.93)	0.02		
AMC								
≥0.5 vs <0.5*10^9^/L	2.35 (1.47–3.74)	<0.001	2.38 (1.38–4.28)	0.003	1.91 (1.26–2.91)	0.002		
HGB								
≥129.5 vs <129.5 g/L	0.48 (0.23–1.00)	0.05			0.43 (0.2–0.83)	0.01	0.24 (0.09–0.62)	0.003
NLR								
≥2 vs <2	2.30 (1.24–4.28)	0.008			2.05 (1.21–3.49)	0.01		
SII								
≥566 vs <566	2.28 (1.41–3.69)	0.001	3.28 (1.98–5.27)	0.02	1.56 (1.02–2.37)	0.04		
ΔNLR%								
≥-0.1 vs <-0.1	2.86 (1.38–5.93)	0.005	2.21 (1.03–4.74)	0.04	2.54 (1.38–4.71)	0.002	2.32 (1.24–4.32)	0.008
ΔSII%								
≥-0.3 vs <-0.3	2.82 (1.25–6.38)	0.01			2.48 (1.27–4.81)	0.006		

WBC, white blood cell count; ALC, absolute lymphocyte count; AMC, absolute monocyte count; ANC, absolute neutrophil count; HGB, hemoglobin; ICB, immune checkpoint blockade; KPS, Karnofsky Performance Status; NLR, neutrophil-lymphocyte ratio; SII, systemic immune-inflammation index; HR, hazard ratio; CI, confidence interval; BMI, body-mass index; LDH, lactate dehydrogenase.

**Figure 1:**
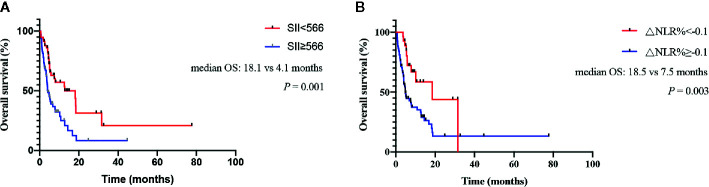
Kaplan-Meier curves for overall survival (OS) of pancreatic cancer (PC) patients treated with immune checkpoint blockade (ICB) therapy. **(A)** Association between baseline systemic immune-inflammation index (SII) and OS. **(B)** Association between neutrophil-lymphocyte ratio (NLR) changes and OS.

The median OS was 4.1 (2.7-5.4) months for patients with baseline SII ≥ 566 vs 18.1 (4.5–31.8) months for patients with SII < 566 (log rank, 11.975; *P*=0.001; **Figure 1A**). For patients with ΔNLR% ≥ -0.1, the median OS was 7.5 (3.9–11.1) months compared with 18.5 (1.4–35.5) months for patients with ΔNLR% < -0.1 (log rank, 8.738; *P*=0.003; **Figure 1B**).

#### PFS

In univariate Cox regression model analysis, a couple of factors, such as tumor stage, KPS scores, ANC, SII, NLR, ΔNLR%, and ΔSII% were all significantly associated with PFS (details in **Table 2**); while in multivariate analysis, only elevated ANC (HR=3.07; 95% CI:1.69–5.60; *P*<0.001), ΔNLR% (HR=2.32; 95% CI: 1.24–4.32; *P*=0.008), and lowered HGB (HR=0.24; 95% CI:0.09–0.62; *P*=0.003) were significantly correlated with an increased risk of progression (**Table 2**). Moreover, since both ΔNLR% and HGB were significant prognostic factors for PFS, we evaluated the significance of ΔNLR%/HGB. Multivariate Cox regression analysis showed that an increased ΔNLR%/HGB was significantly associated with an increased risk of progression (HR=2.32; 95% CI: 1.24–4.32; *P*=0.008).

**Figure 2:**
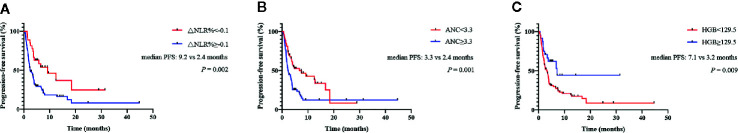
Kaplan-Meier curves for progression-free survival (PFS) of pancreatic cancer (PC) patients receiving immune checkpoint blockade (ICB) therapy. **(A)** Association between neutrophil-lymphocyte ratio (NLR) changes and progression-free survival (PFS). **(B)** Association between baseline absolute neutrophil count (ANC) and PFS. **(C)** Association between baseline hemoglobin (HGB) and PFS.

For patients with ΔNLR% ≥ -0.1, the median PFS was 2.4 (1.2–3.6) months compared with 9.2 (2.5–15.9) months for patients with ΔNLR% < -0.1 (log rank, 19.522; *P*= 0.002; **Figure 2A**). The median PFS was 2.4 (1.7–3.0) months for patients with baseline ANC ≥ 3.3 vs 6.7 (1.1–12.2) months for patients with ANC < 3.3 (log rank, 10.156; *P*= 0.001; **Figure 2B**). For patients with HGB ≥ 129.5, the median PFS was 7.1 months compared with 3.2 (2.2–4.2) months for patients with HGB < 129.5 (log rank, 6.801; *P*=0.009; **Figure 2C**).

In order to investigate whether the combined chemotherapies affected the systemic immunity, we categorized the patients into five groups based on their combined chemotherapies they received: gemcitabine, paclitaxel group, 5-fluorouracil group, cisplatin group and targeted therapy group. The descriptive data of the NLR and SII values in these groups are listed in **Supplementary Table 1**. We compared their NLR and SII values after two cycles of treatment using analysis of variance (ANOVA), and the results showed that the average NLR and SII values between these groups weren’t significantly different (*P*=0.181 and *P*= 0.281, respectively).

### Association Between Blood Biomarkers and Prognosis When ICB Was the First-Line Treatment

In order to eliminate influence from other treatments on clinical outcomes, we then made a subgroup analysis, focusing on PC patients receiving ICB combined with chemotherapies or radiotherapies as first-line treatment. Results showed that 45 PC patients received ICB therapy combined with chemotherapies or radiotherapies as first-line treatment, and their clinical characteristics are presented in **Table 3**. Their median age at the first dose of ICB therapy was 62 years (range 42–84), and 66.7% were male. By the end of December 2019, 14 patients (31.1%) were confirmed to be alive, 16 (35.6%) had confirmed disease progression, and 10 (22.2%) were lost to follow-up. The median OS was 6.6 months (0.9–31.4) and median PFS was 4.6 months (0.6–31.4).

**Table 3:** Baseline characteristics of PC patients receiving ICB as first-line treatment.

Characteristic	Total (N = 45)
**Age, median (range)**	62 (42–84)
**Gender, N (%)**	
Male	30 (66.7)
Female	15 (33.3)
**Tumor stage at diagnosis, *N* (%)**	
locally advanced	7 (15.6)
metastatic	38 (84.4)
**Tumor location, *N* (%)**	
head	26 (57.8)
body-tail	16 (35.6)
both	1 (2.2)
unavailable	2 (4.4)
**CA19-9, u/ml, *N*(%)**	
<1,000	23 (51.1)
≥1,000	18 (40.0)
Unavailable	4 (8.9)
**Previous surgery, *N* (%)**	
yes	13 (28.9)
no	32 (71.1)
**KPS, *N* (%)**	
≥90	35 (77.8)
80	8 (17.8)
70	1 (2.2)
≤60	1 (2.2)
**BMI, *N* (%)**	
<18.5	9 (20.0)
18.5≤BMI<24	28 (62.2)
24≤BMI<28	6 (13.3)
≥28	0 (0)
Unavailable	2 (4.5)
**LDH, *N* (%)**	
normal	30 (66.7)
abnormal	14 (31.1)
unavailable	1 (2.2)

ICB, immune checkpoint blockade; KPS, Karnofsky Performance Status; LDH, lactate dehydrogenase; BMI, body-mass index.

#### OS

**Figure 3:**
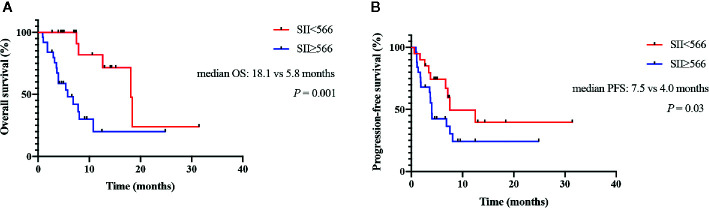
Kaplan-Meier curves for survival of pancreatic cancer (PC) patients receiving immune checkpoint blockade (ICB) combined with chemotherapies as the first-line treatment. **(A)** Association between baseline systemic immune-inflammation index (SII) and overall survival (OS). **(B)** Association between baseline SII and progression-free survival (PFS).

In univariate Cox regression model analysis, ALC, AMC, SII, NLR and ΔSII% were all significantly associated with OS (details in **Table 4**); while in multivariate analysis, only elevated SII (HR=8.06; 95% CI:1.71–37.86; *P*=0.01) was significantly correlated with an increased risk of death (**Table 4**). The median OS was 5.8 (2.2–9.4) months for patients with baseline SII ≥ 566 vs 18.1 (13.0–23.3) months for patients with SII < 566 (log rank, 10.563; *P*=0.001; **Figure 3A**).

**Table 4:** Univariate and multivariate Cox regression analyses of prognostic factors associated with prognosis when ICB as first-line treatment.

Factors	Overall survival				Progression-free survival			
	Univariate analysis		Multivariate analysis		Univariate analysis		Multivariate analysis	
	HR (95% CI)	*P*	HR (95% CI)	*P*	HR (95% CI)	*P*	HR (95% CI)	*P*
Gender								
male vs female	0.55 (0.22–1.34)	0.18			0.75 (0.34–1.67)	0.48		
Age								
≥ 65 vs < 65	0.53 (0.18–1.57)	0.24			0.92 (0.40–2.10)	0.84		
KPS scores		0.06				0.01		
70 or 80 vs ≥ 90	0.72 (0.24–2.18)				1.13 (0.45–2.83)			
≤ 60 vs ≥ 90	7.07 (0.89–66.42)				14.54 (1.50–141.10)			
BMI		0.76				0.70		
BMI < 18.5 vs 18.5 ≤ BMI < 24	1.44 (0.51–4.07)				1.48 (0.58–3.81)			
BMI ≥ 24 vs 18.5 ≤ BMI < 24	0.93 (0.26–3.31)				1.23 (0.41–3.69)			
LDH								
abnormal vs normal	1.13 (0.44–2.87)	0.80			1.55 (0.70–3.44)	0.28		
Tumor stage								
metastatic vs locally advanced	2.32 (0.54–10.00)	0.25			3.57 (0.84–15.12)	0.07		
CA19–9								
≥1000 vs <1000 u/ml	1.63 (0.61–4.34)	0.33			1.28 (0.56–2.91)	0.56		
Previous surgery								
no vs yes	1.58 (0.60–4.12)	0.35			1.48 (0.64–3.41)	0.36		
WBC								
≥4.9 vs <4.9*10^9^/L	1.74 (0.63–4.79)	0.28			1.44 (0.62–3.31)	0.39		
ANC								
≥3.3 vs <3.3*10^9^/L	1.95 (0.75–5.03)	0.16			1.53 (0.68–3.41)	0.30		
ALC								
≥1.1 vs <1.1*10^9^/L	0.32 (0.12–0.83)	0.01			0.41 (0.18–0.93)	0.03		
AMC								
≥0.5 vs <0.5*10^9^/L	2.43 (1.03–5.73)	0.04			1.71 (0.79–3.69)	0.17		
HGB								
≥129.5 vs <129.5 g/L	0.50 (0.17–1.47)	0.20			0.57 (0.23–1.42)	0.22		
NLR								
≥2 vs <2	4.71 (0.63–35.38)	0.10			2.19 (0.66–7.30)	0.19		
SII								
≥566 vs <566	4.83 (1.71–13.62)	0.001	8.06 (1.71–37.86)	0.01	1.82 (1.53–3.99)	0.01	2.84 (1.37–10.38)	0.04
ΔNLR%								
≥-0.1 vs <-0.1	2.86 (0.89–9.18)	0.07			2.03 (0.80–5.17)	0.13		
ΔSII%								
≥-0.3 vs <-0.3	3.35 (1.04–10.78)	0.03			2.17 (0.85–5.51)	0.10		

WBC, white blood cell count; ALC, absolute lymphocyte count; AMC, absolute monocyte count; ANC, absolute neutrophil count; HGB, hemoglobin; ICB, immune checkpoint blockade; KPS, Karnofsky Performance Status; NLR, neutrophil-lymphocyte ratio; SII, systemic immune-inflammation index; HR, hazard ratio; CI, confidence interval; BMI, body-mass index; LDH, lactate dehydrogenase.

#### PFS

In univariate Cox regression model analysis, KPS scores, ALC and SII were all significantly associated with PFS (details in **Table 4**); while in multivariate analysis, only elevated SII (HR=2.84; 95%CI:1.37–10.38; *P*=0.04) were significantly correlated with an increased risk of progression (**Table 4**). The median PFS was 4.0 (3.5–4.4) months for patients with baseline SII ≥ 566 vs 7.5 (8.2–14.8) months for patients with SII < 566 (log rank, 6.274; *P*=0.03; **Figure 3B**).

## Discussion

In the present study, we confirmed the independent and significant association between SII at baseline, NLR changes and clinical outcomes of PC patients treated with ICB therapy. To our best knowledge, this is the first study of systemic inflammation as prognostic biomarkers in clinical outcomes of PC patients receiving ICB therapy, and the results warrant prospective validation in a larger cohort.

Our results revealed that elevated SII was a negative predictor for OS of PC patients treated with ICB and OS/PFS of PC patients receiving ICB combined with chemotherapies as first-line treatment. The prognostic value of SII has been investigated in a couple of solid tumors, including gastrointestinal malignancies (25, 26). For patients with resectable pancreatic ductal adenocarcinoma (PDAC), SII is superior to NLR and PLR for predicting OS (27). In addition, the prognostic significance of baseline SII was also validated in advanced PC patients with both normal and elevated CA19-9 levels (28). For patients with resected pancreatic cancer, the elevated SII after neoadjuvant therapy was identified as an independent negative predictor of OS (29). In another study, Mohammad et al. analyzed 590 patients with resectable PDAC (30). SII >900 [hazard ratio (HR) 2.32, 95% confidence interval (CI) 1.55–3.48] was identified as an independent predictor of cancer-specific survival. Moreover, there have been studies reporting SII significance in survival of patients undergoing immunotherapy (31). Ugo De Giorgi and colleagues validated SII and BMI were critical prognostic factors for OS in patients with renal cell carcinoma (RCC) treated with nivolumab (32). Similarly, SII, NLR and platelet-to-lymphocyte ratio (PLR) were identified as promising prognostic predictors for patients with metastatic non-small cell lung cancer (NSCLC) patients treated with nivolumab (33). Based on the definition of SII, elevated SII may be attributed to thrombocythemia, neutrophilia, and lymphopenia. It has been reported that neutrophilia and thrombocythemia are associated with pro-tumorigenic functions (34–36). Neutrophils are able to promote proliferation and metastasis of tumor cells and help with undermining immune surveillance (37). Additionally, neutrophils also revive the senescent cancer cell and suppress T cell activation to promote immune evasion (38). Platelets keep circulating tumor cells (CTCs) from shearing stresses in vessels and inducing CTC epithelial-mesenchymal transition (39). Generally speaking, lymphopenia implies the compromise of the immune system. Lymphopenia has also been reported in pancreatic cancer (40) and identified to be correlated with poor clinical outcomes in several malignant tumors (41). To some extent, our results reveal the roles of neutrophils, platelets, and lymphocytes in cancer immunology.

Aside from the baseline peripheral blood biomarkers, we believe that the change of biomarkers during immunotherapy treatment would be more informative and indicate a more direct effect from the immunotherapy treatment itself. Our results showed that great relative NLR changes after two doses of ICB, regardless of baseline values, were associated with a shorter PFS for PC patients. In recent years, the prognostic significance of baseline NLR in PC patients has been extensively explored, and higher NLR was always associated with poor clinical outcomes (42–46). Meanwhile, the changes of NLR during clinical courses have also gained much attention. In gastric cancer, NLR changes after the nivolumab monotherapy were associated with patient survival (47). For metastatic RCC patients treated with ICB, the decrease of NLR at 6 weeks was correlated with significantly improved outcomes (48). Intriguingly, another study revealed that the non-linear changes of NLR were correlated with clinical outcomes of cancer patients (49). In a cohort of various advanced cancers, a moderate decrease in NLR during ICB treatment was associated with the longest survival, whereas patients with a significant decrease or increase in NLR had shorter survival. The dynamic NLR changes may imply the effects of ICB therapy on immune system regardless of baseline values. Because of the inexpensive and readily available feature of NLR, it is quite convenient to calculate and monitor NLR changes during clinical courses.

In our Cox multivariate analysis, the results showed that lower HGB was significantly associated with shortened PFS for PC patients treated with ICB. There have been a couple of studies investigating the association between HGB and clinical outcomes of PC patients. A previous study showed that HBG was an independent factor for OS of metastatic PDAC patients (50). In addition, the study by Ruiz-Tovar et al. demonstrated that the preoperative levels of hemoglobin under 12 g/dl was associated with worse survival (*P*= 0.0006) (51). In Japanese PC patients who received gemcitabine monotherapy as first-line chemotherapy, serum hemoglobin level≥10 g/dl was identified as an independent favorable prognostic factor (*P* = 0.01) (52). Another retrospective study reported that preoperative levels of hemoglobin <12 g/dl was associated with poor survival in PC patients (*P*= 0.0006) (53). Moreover, in patients with non-metastatic locally advanced PC treated at M.D. Anderson Cancer Center (Houston, Texas), analysis showed that HGB and KPS scores were independent prognostic factors for OS (54). It is consistent with our clinical experience that the low level of HGB may predict poor clinical outcomes, but the mechanisms underlying their association have barely been investigated yet. Anemia may be connected with tumor hypoxia, and researchers have found that neoplastic cells acquire resistance to radiochemotherapy in hypoxic conditions (55). Tumor hypoxia has been found to be associated with a higher probability of distant metastases (56). Additionally, another study observed that anemia was associated with severe deficiency of CD8^+^ T cell responses against pathogens in treatment-naive mice bearing large tumors, and an immunosuppressive CD45^+^ erythroid progenitor cell (EPC) population was detected in cancer patients with anemia (57). This may reveal the effects of cancer on hematopoiesis and adoptive immunity. Moreover, we showed that an increased ΔNLR%/HGB was significantly associated with an increased risk of progression of pancreatic cancer. As ΔNLR%/HGB is derived from ΔNLR% and HGB, it takes into account both the systemic inflammation and hypoxic status of cancer patients simultaneously. Thus, the prognostic significance of ΔNLR%/HGB may be stronger and needs further validation in future prospective studies.

Limitations of this study include the fact that we performed a retrospective analysis in a single center and the sample was relatively small. Moreover, the ICB therapy patients have received consists of different kinds of monoclonal antibodies, which might weaken the interpretation of results. In addition, the optimal cutoff values of prognostic factors have no consensus now. We obtain our best cutoff values using the ROC curve, and their significance should be validated in other independent cohorts of cancer patients.

To sum up, our research results show that the baseline SII and NLR changes are independent prognostic factors of OS/PFS for PC patients treated with ICB therapy, and the baseline SII is also significantly associated with OS/PFS of patients receiving ICB combined with chemotherapies as first-line treatment. Their potential of helping with patient stratification and clinical decision making should be developed accordingly. The predictive ability of peripheral blood biomarkers for prognosis of PC patients receiving ICB therapy should be further confirmed in subsequent larger prospective studies.

## Data Availability Statement

The original contributions presented in the study are included in the article/**Supplementary Material**. Further inquiries can be directed to the corresponding author.

## Ethics Statement

The studies involving human participants were reviewed and approved by the Ethics Committee of Chinese PLA general hospital. The patients/participants provided their written informed consent to participate in this study.

## Author Contributions

JS, XH, HZ, and YH contributed to the study concepts and study design. Data acquisition was performed by JS and XH. HT, XL, FY, GC, LW, and JM were involved in the identification and selection of patients. All the authors contributed to the quality control data, analysis, and interpretation of data. The statistical analysis was performed by JS. The manuscript preparation was performed by JS and XH and edited by YH. All authors contributed to the article and approved the submitted version.

## Funding

This work was supported by a grant from the Military Health special research project [19BJZ15 to YH]; and the National Natural Science Foundation of China [31900627 to HZ, 81902917 to XH].

## Conflict of Interest

The authors declare that the research was conducted in the absence of any commercial or financial relationships that could be construed as a potential conflict of interest.
