# Supplementary material for: Systemic Immune-Inflammation Index and Changes of Neutrophil-Lymphocyte Ratio as Prognostic Biomarkers for Patients With Pancreatic Cancer Treated With Immune Checkpoint Blockade
Source: Front Oncol. 2021 Feb 24;11:585271. doi: 10.3389/fonc.2021.585271 (PMC7943876; doi:10.3389/fonc.2021.585271)
Supplement: Supplementary file 1 [file Table_1.docx]

**Supplementary Table 1 Average NLR and SII values after two cycles of combined chemotherapies**

| Chemotherapy groups | Patient number | NLR | | SII | |
| --- | --- | --- | --- | --- | --- |
|  |  | Average | SD | Average | SD |
| Gemcitabine group | 25 | 3.47 | 2.05 | 919.77 | 679.33 |
| Paclitaxel group | 37 | 5.51 | 7.48 | 1150.36 | 1447.62 |
| 5-fluorouracil group | 22 | 4.91 | 6.52 | 848.21 | 1034.69 |
| Cisplatin group | 7 | 1.60 | 0.76 | 300.60 | 183.34 |
| Targeted therapy group | 27 | 7.44 | 9.64 | 1373.37 | 1289.49 |

Abbreviations: NLR, neutrophil-lymphocyte ratio; SII, systemic immune-inflammation index; SD, standard deviation.
